# Berbamine inhibits Japanese encephalitis virus (JEV) infection by compromising TPRMLs-mediated endolysosomal trafficking of low-density lipoprotein receptor (LDLR)

**DOI:** 10.1080/22221751.2021.1941276

**Published:** 2021-06-26

**Authors:** Lihong Huang, Huanan Li, Zuodong Ye, Qiang Xu, Qiang Fu, Wei Sun, Wenbao Qi, Jianbo Yue

**Affiliations:** a City University of Hong Kong Shenzhen Research Institute, Shenzhen, People’s Republic of China; b Department of Biomedical Sciences, City University of Hong Kong, Hong Kong, People’s Republic of China; c College of Veterinary Medicine, South China Agricultural University, Guangzhou, People’s Republic of China; d College of Veterinary Medicine, Xinjiang Agricultural University, Urumqi, People’s Republic of China; e City University of Hong Kong Chengdu Research Institute, Chengdu, People’s Republic of China

**Keywords:** Berbamine, flavivirus, JEV, LDLR, Ca2+, TRPMLs, extracellular vesicles

## Abstract

Japanese encephalitis virus (JEV), a member of the *Flavivirus* genus, is an important pathogen that causes human and animal infectious diseases in Asia. So far, no effective antiviral agents are available to treat JEV infection. Here, we found that LDLR is a host factor required for JEV entry. Berbamine significantly decreases the level of LDLR at the plasma membrane by inducing the secretion of LDLR via extracellular vesicles (EVs), thereby inhibiting JEV infection. Mechanistically, berbamine blocks TRPMLs (Ca^2+^ permeable non-selective cation channels in endosomes and lysosomes) to compromise the endolysosomal trafficking of LDLR. This leads to the increased secretion of LDLR via EVs and the concomitant decrease in its level at the plasma membrane, thereby rendering cells resistant to JEV infection. Berbamine also protects mice from the lethal challenge of JEV. In summary, these results indicate that berbamine is an effective anti-JEV agent by preventing JEV entry.

## Introduction

*Flaviviruses* such as Japanese encephalitis virus (JEV), dengue virus (DENV), Zika virus (ZIKV), West Nile virus (WNV), yellow fever virus (YFV), and tick-borne encephalitis virus (TBEV) are among the most significant human pathogens. They are mainly transmitted to humans through infected mosquitoes and ticks [1–4]. Japanese encephalitis cases have been mainly reported in Asian countries, especially in East and Southeast Asia [5]. Although a large proportion of human cases of JEV infection are asymptomatic, the features of human infection with JEV range from mild fever to severe haemorrhagic and encephalitic manifestations or even death. Over the past few decades, inactivated and living-attenuated JEVs have been successfully used in producing vaccines in many countries, and immunization programs have markedly decreased the burden of disease. However, it was estimated that annually JEV still affects about 68,000 people and results in 10,000-15,000 deaths, with 30%−50% of survivors showing lifelong neurological sequelae [5]. Currently, patients with serious JEV infection only receive supportive care, including intravenous fluids, hospitalization, respiratory support, and the prevention of secondary infections. Therefore, there is an urgent need for safe and effective anti-JEV agents.

The endosomal trafficking system is comprised of a series of dynamically interconverted membrane-enclosed vesicular structures, including early endosome (EE), multivesicular body (MVB), and late endosome (LE)[6]. When EEs mature to MVBs, the inward invagination of the membrane of MVBs forms the intraluminal vesicles (ILVs) inside the lumen of MVBs[7]. During this process, membrane tetraspanins, including CD81, CD63, and CD9, are incorporated into the invaginated membrane, while some cytosolic contents, e.g. proteins (TSG101, ALIX, HSP70, HSP90, *etc.*), nucleic acids (RNA and DNA), metabolites, and amino acids, are enclosed inside ILVs[7]. MVBs can either fuse with lysosomes for degradation or fuse with the plasma membrane to release its luminal ILVs. These released ILVs are called exosomes, a subset of extracellular vesicles (EVs) with sizes ranging from 30–150 nm. Another subset of EVs is the vesicles directly budding from cell plasma membrane with size ranging from 50–1000 nm. The cell–cell communications via the release and uptake of exosomes have been implicated in a number of physiological and pathological processes [8].

Low-density lipoprotein receptor (LDLR), a single-chain single-pass transmembrane glycoprotein, is the receptor for low-density lipoprotein (LDL), and is responsible for lipoproteins trafficking and lipid metabolism [9,10]. The internalized receptor–ligand complexes are firstly clustered into coated pits, followed by transport to early and late endosomes. LDLR is ubiquitously expressed, and the LRLR-ligand complexes are internalized into cells by endocytosis every 10 min [11,12]. LDLR has been reported to function as a receptor for the hepatitis C virus (HCV), human rhinoviruses, and vesicular stomatitis viruses (VSV) [13–17]. Here, we found that LDLR is involved in JEV entry into host cells and can bind to JEV-E, suggesting that LDLR is a potential cellular receptor for JEV. Moreover, we found that berbamine, a bis-benzylisoquinoline alkaloid isolated from herbs and a known calcium channel or signaling inhibitor, significantly decreases the LDLR at the plasma membrane, thereby rendering cells resistant to JEV infection. We further found that berbamine inhibits the transient receptor potential membrane channel mucolipin (TRPML) family to impair the lysosome function, which in turn compromises the endolysosomal trafficking of LDLR. This leads to the increased secretion of LDLR via extracellular vesicles (EVs) and the concomitant decrease of its levels at the plasma membrane, thereby preventing JEV from entering host cells.

## Materials and methods

*Cell culture and virus propagation-* HeLa, A549, Vero, BHK-21, or 4T1 cells were maintained in DMEM (Gibco, 12800082) containing 10% fetal bovine serum (Gibco, 10500064) and 100 U/ml of penicillin/streptomycin. The JEV SA14-14-2 strain was amplified from BHK-21 cells, which was maintained in DMEM containing 2% fetal bovine serum and 100 U/ml of penicillin/streptomycin.

*Immunofluorescence staining-* Cells were fixed with 4% paraformaldehyde (PFA) in warm 1x PBS at room temperature (RT) for 15 min. After rinsed with PBS three times, cells were blocked with PBS containing 5% normal donkey serum and 0.3% Triton™ X-100 at RT for 1 h, and then incubated with primary antibody at 4°C overnight, followed by the appropriate fluorescent secondary antibody. To label the receptors on the plasma membrane, live cells were incubated with the primary antibody in PBS (+1% BSA) on ice for 90 min, followed by incubation with the fluorescent secondary antibody on ice. Images were captured with Carl Zeiss LSM 880 confocal microscopes using a 63×oil objective lens. The primary antibodies used in these experiments are listed in Table S1.

*In situ RNA hybridization- In situ* RNA hybridization was performed with an RNAscope® Multiplex Fluorescent kit (Advanced Cell Diagnostics, 320851) by following the manufacturer’s instructions. In brief, cells plated on coverslips were fixed with 4% PFA, permeabilized, and incubated with a specific RNA probe targeting the JEV (ACD, 435551) viral genome for 2 h at 40°C. Then, up to four signal amplification systems were used to detect the target RNA. After RNA hybridization, the cells were subjected to immunofluorescence staining as described above.

*Western blot analysis-* The Bradford assay (Bio-RAD) was performed to measure the protein concentration of cell lysates. Equal amounts of cell lysates per sample were loaded onto 8%−12% SDS-PAGE gels for electrophoresis. The proteins were then transferred to a PVDF membrane (Millipore), blocked with 5% non-fat milk, and blotted with primary and secondary antibodies. The primary antibodies used for immunoblotting are listed in **Table S2**.

*Cytotoxicity assay-* BHK-21, A549, or HeLa cells, plated in 96-well plates (Corning, 3603), were treated with different concentrations of berbamine dihydrochloride (sigma, 547190) for 24 h. The cells were then stained with propidium iodide (PI; Invitrogen, P3566) and Hoechst 33258 (Invitrogen, H3570), and images were acquired using a CellInsight CX7 High-Content Screening platform with a 10× objective lens. Quantification of the dead (PI-positive) cells was performed with HCS Studio™ 3.0 (Thermo Fisher). The half-maximal cytotoxicity concentration (CC_50_) was calculated via Graphpad Prism 5.

*The antivirus activity of drugs-* A549 cells were pretreated with chemicals at the indicated concentrations for 1 h, and infected with ∼1 MOI of JEV. At 48 h post-infection, cells were fixed with 4% PFA, stained with an anti-dsRNA antibody, and subjected to the high content screening platform to acquire fluorescence images. The percentage of infected cells was determined using HCS Studio™ 3.0. The 50% of maximal effect (EC_50_) was calculated with Graphpad Prism 5.

*JEV and LDL competitive assay-* Firstly, A549 cells grown in a 96-well plate (Corning, 3603) were serum-starved overnight by using Fluorobrite DMEM medium (Cat. No. A1896702) plus 0.3% BSA. Then, cells were incubated with the indicated dose of JEV on ice for 1 h, followed by incubation at 37°C for 10 min to allow the internalization of bound JEV. Subsequently, cells were incubated with fluorescently labeled LDL (Dil LDL, Cat. No. L3482) on ice for 1 h before being placed on 37 ˚C for 30 min to start the internalization of bound Dil LDL. Lastly, cells were fixed with 4% PFA and subjected to DAPI staining and high-content screening to collect images and analyze the uptake of Dil LDL.

*LDL uptake assay-* A549 cells grown in 96-well plate were-serum starved overnight by using Fluorobrite DMEM medium plus 0.3% BSA. Then, cells were pretreated with DMSO, Heparin (250 μg/mL), or berbamine (10 or 25 μM) for 3 h, followed by incubation with Dil LDL on ice for 1 h. Afterward, the cells were incubated at 37 ˚C for 30 min to allow the internalization of Dil LDL. Lastly, cells were fixed with 4% PFA and subjected to DAPI staining and high-content screening to collect images and analyze the uptake of Dil LDL.

*Analysis of size distribution and concentration of extracellular vesicles-* A549 cells grown in 6-well dishes at about 80% confluency were rinsed with PBS twice and changed with 1.2 ml of an EV-depleted complete medium containing DMSO or berbamine (50 μM). Six hours later, supernatants in each well were collected and subjected to sequential centrifugation at different centrifugal forces (g) to remove intact cells, dead cells, and cell debris. Finally, the supernatants containing EVs were analyzed with a nanoparticle tracking analyzer (NanoSight NS300, Malvern) to determine the concentrations and size distribution.

*Purification of EVs from the culture medium-* A549 cells were grown in 15-cm dishes to ∼80% confluency. The cells were then rinsed with PBS and incubated in an EV-depleted complete medium containing DMSO or berbamine (25 μM) for 48 h. The supernatant was collected and subjected to sequential centrifugation steps at different centrifugal forces (g) to remove the intact cells, dead cells, or cell debris. After each centrifugation, the supernatant was transferred into a new 50 ml tube, and the pellet was discarded. Finally, the supernatant was subjected to ultracentrifugation at 120,000 × g for 90 min, and the pellet (now containing EVs) was washed with PBS and subjected to another ultracentrifugation at 120,000 × g for 90 min. Finally, the exosome pellet was collected and used for immunoblot analysis.

*Intracellular Ca^2+^ measurements-* HeLa cells were grown in 24-well plates to ∼80% confluency. The cells were then loaded with HBSS (Gibco, 14025092) containing 4 μM Fura-2 AM (Invitrogen, F1221) and 0.4% Pluronic™ F-127 (Invitrogen, P3000MP) at room temperature for 30 min. The cells were washed with Ca^2+^-free HBSS containing 2 mM EGTA, and incubated in Ca^2+^-free HBSS in the presence or absence of berbamine (10 μM) at room temperature for another 30 min. Fluorescence images were acquired at 3 s intervals by alternate excitation at 340 and 380 nm with emission at 510 nm using a Nikon Eclipse Ti-S Calcium imaging system. Approximately 1 min after live-cell imaging, 200 μM GPN (Abcam, ab145914), or 25 μM ML-SA1 (Tocris Bioscience, 4746) was added to the cells to trigger Ca^2+^ release from the lysosomes.

*Small interference RNA (siRNA)-* Cells were transfected with siRNAs against respective genes (**Table S3**) using Lipofectamine 3000 according to the manufacturer’s instructions. The knockdown efficiency was validated by immunoblot analysis or qRT-PCR.

*The anti-JEV activity of berbamine in mice*- The anti-JEV activity of berbamine was performed in BALB/c mice as described previously [18]. Briefly, 3–4-week BALB/c mice were randomly divided into four groups (eight mice per group): an uninfected and PBS-treated group, an uninfected and berbamine-treated group, a JEV-infected and PBS-treated group, and a JEV-infected and berbamine-treated group. Mice were first injected intraperitoneally with PBS or 15 mg/kg of bodyweight of berbamine. 6 h later, mice were infected intraperitoneally with 10^7^ TCID_50_ of JEV (SA14 virus strain). Thereafter, mice were treated with PBS or berbamine (15 mg/kg) twice per day for 14 days. The mice were monitored daily for morbidity and mortality. The mice that showed severe neurological signs of disease were euthanized. All animal studies were performed in B3 level laboratories by strictly following the safety and animal ethics guidelines of the university and government.

*Statistical analysis-* Data are presented as mean ± S.E.M. Statistically significant differences were determined by the Student’s t-test, and *P* < 0.05 was considered to be statistically significant.

## Results

**Figure 1.**
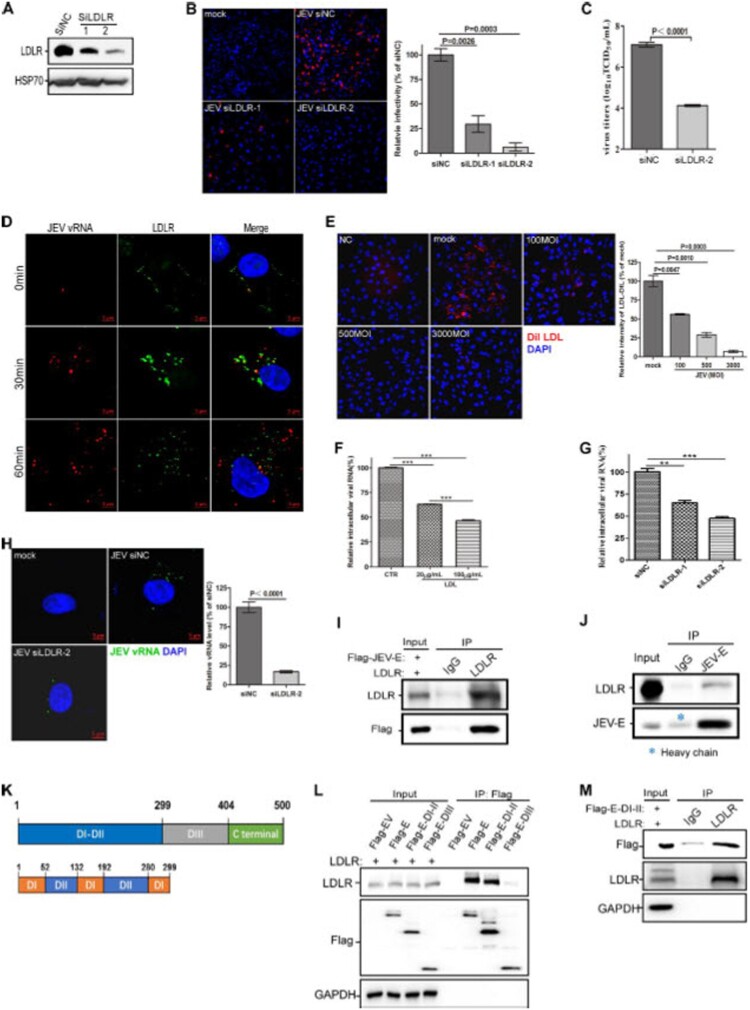
**LDLR is required for JEV internalization.** (**A**) Knockdown efficiency of LDLR in A549 cells by LDLR siRNAs. (**B**, **C**) A549 cells were transfected with LDLR siRNAs and were then infected with ∼1 MOI of JEV, followed by immunostaining against dsRNA at 48 h.p.i. (**B**), or titer measurement by TCID_50_ assay at 24 h.p.i. (**C**). (**D**) A549 cells were incubated with an anti-LDLR primary antibody and an Alexa Fluor 488-tagged secondary antibody (green) on ice, before being infected with ∼50 MOI of JEV on ice. Subsequently, the cells were incubated at 37 ˚C for indicated times, followed by in situ RNA hybridization for detecting the JEV RNA genome (red). (**E**) A549 cells were incubated with an increasing amount of JEV on ice for 1 h, followed by incubation at 37 ˚C for 10 min to allow the endocytosis of bound JEV. Then, cells were incubated with fluorescently labeled LDL (Dil LDL) on ice for 1 h before being placed on 37 ˚C for 30 min to start the internalization of bound Dil LDL. (**F**) A549 cells were incubated with LDL (0, 20 μg/ml or 100 μg/ml) at 37 ˚C for 30 min before incubating with ∼ 50 MOI of JEV on ice for another 1 h. The cells were then incubated at 37 ˚C for 30 min, and qPCR was performed to quantify the entry-level of JEV. (**G**) A549 cells were transfected with LDLR siRNAs, followed by incubation with ∼ 50 MOI of JEV on ice for 1 h. Cells were then incubated at 37 ˚C for 30 min, and qPCR was performed to quantify the entry-level of JEV. (**H**) A549 cells were transfected with LDLR siRNAs, followed by 50 MOI of JEV infection for 80 min. Then, *in situ* RNA hybridization was performed to detect the internalization of JEV. (**I**) HEK293T cells were transfected with JEV-E (tagged with Flag) and LDLR. The interaction between JEV-E and LDLR was confirmed by a co-IP assay. Bound proteins were eluted and analyzed by immunoblot with LDLR and Flag-tag antibodies. (**J**) The cell lysates from HEK293T cells that overexpressed LDLR were incubated with Rabbit IgG or JEV-E antibody, followed by immunoblot analysis. (**K**) Schematic diagram of JEV-E structure. (**L**) The empty vector (Flag-EV), a full-length JEV-E (Flag-E), first and second domain of JEV-E (Flag-E-DI-II), or the third domain of JEV-E (Flag-E-DIII) was transfected with LDLR into HEK293T cells. Cell lysates were subjected to immunoprecipitation using anti-Flag Magnetic Beads and immunoblot analysis. (**M**) HEK293T cells were co-transfected with Flag-E-DI-II and LDLR. Cell lysates were then subjected to immunoprecipitation with Rabbit IgG or anti-LDLR antibody, followed by immunoblot analysis. The blots, images, and graphs represent data from three independent experiments. The difference between the two groups was analyzed using a two-tailed Student's t-test, *P*<0.05 was considered statistically significant.

***LDLR is required for JEV internalization.*** Since LDLR has been reported to function as a receptor for HCV and VSV, we assessed whether LDLR is involved in JEV infection. We found that LDLR knockdown markedly decreased JEV infection in A549 cells (Figure 1(A and B)). Moreover, LDLR knockdown significantly impaired JEV propagation in A549 cells (Figure 1(C)). In addition, we examined whether JEV infection triggers the internalization of LDLR. Thus, live A549 cells were incubated on ice with the anti-LDLR antibody and an Alexa Fluor 488-tagged secondary antibody to label the cell surface LDLR, followed by JEV incubation on ice for 1 h. Thereafter, cells were incubated at 37 ˚C to trigger the internalization of the virus and/or LDLR-antibody complex, and subjected to *in situ* RNA hybridization to detect the vRNA of the JEV at the indicated time points. We showed that the internalized LDLR-positive endosomes exhibited strong colocalization with the vRNA particles by 30 min after virus infection (middle panel in Figure 1(D)). The vRNA particles then became dissociated from the LDLR-positive endosomes by 60 min after virus infection (lower panel in Figure 1(D)), and we suspect that this is likely due to the release of viral genome RNA from the endosomes.

Since LDLR is endocytosed together with JEV virion particles, we investigated whether JEV competes with LDL (the LDLR ligand) for binding to LDLR. Briefly, A549 cells were first incubated with an increasing amount of JEV on ice for 1 h, followed by incubation at 37 ˚C for 10 min to allow the endocytosis of the bound JEV. Thereafter, cells were incubated with fluorescently labeled LDL (Dil LDL) on ice for 1 h followed by incubation at 37 ˚C for another 30 min to initiate the internalization of the bound Dil LDL. As expected, JEV infection significantly decreased LDL internalization in a concentration-dependent manner (Figure 1(E)), further confirming that LDLR is transported into cells during JEV internalization. Likewise, preincubation with LDL significantly inhibited JEV internalization (Figure 1(F)), indicating that LDLR is required for the entry of JEV into host cells. To further confirm LDLR is required for JEV internalization, control or LDLR-knockdown A549 cells were incubated with JEV on ice for 1 h, and were then incubated with a warm medium at 37 ˚C for the indicated time followed by qPCR (Figure 1(G)) or *in situ* RNA hybridization (Figure 1(H)) to detect the JEV vRNA. As expected, LDLR knockdown significantly inhibited the levels of JEV vRNA (Figure 1(G and H)). In summary, these results indicate that LDLR is required for JEV entry into host cells.

Subsequently, we performed the coimmunoprecipitation (co-IP) to determine whether LDLR interacts directly with JEV envelope protein (E), the viral protein responsible for cellular attachment and receptor binding. In HEK293T cells overexpressing Flag-tagged JEV-E and LDLR, the anti-LDLR antibody brought down both LDLR and JEV-E (Figure 1(I)). Likewise, the anti-JEV-E antibody pulled down both JEV-E and LDLR (Figure 1(J)). The ectodomain of JEV-E protein contains three domains: DI, DII and DIII, and DII is composed of two extended loops that protrude from DI **(**Figure 1(K)**)**. We, thus, truncated the E protein into DI-DII and DIII to determine which fragment of JEV-E interacts with LDLR. We showed that the DI-DII of E protein is responsible for interacting with LDLR (Figure 1(L and M)). These data suggest that LDLR is a potential host receptor for JEV entry.

**Figure 2.**
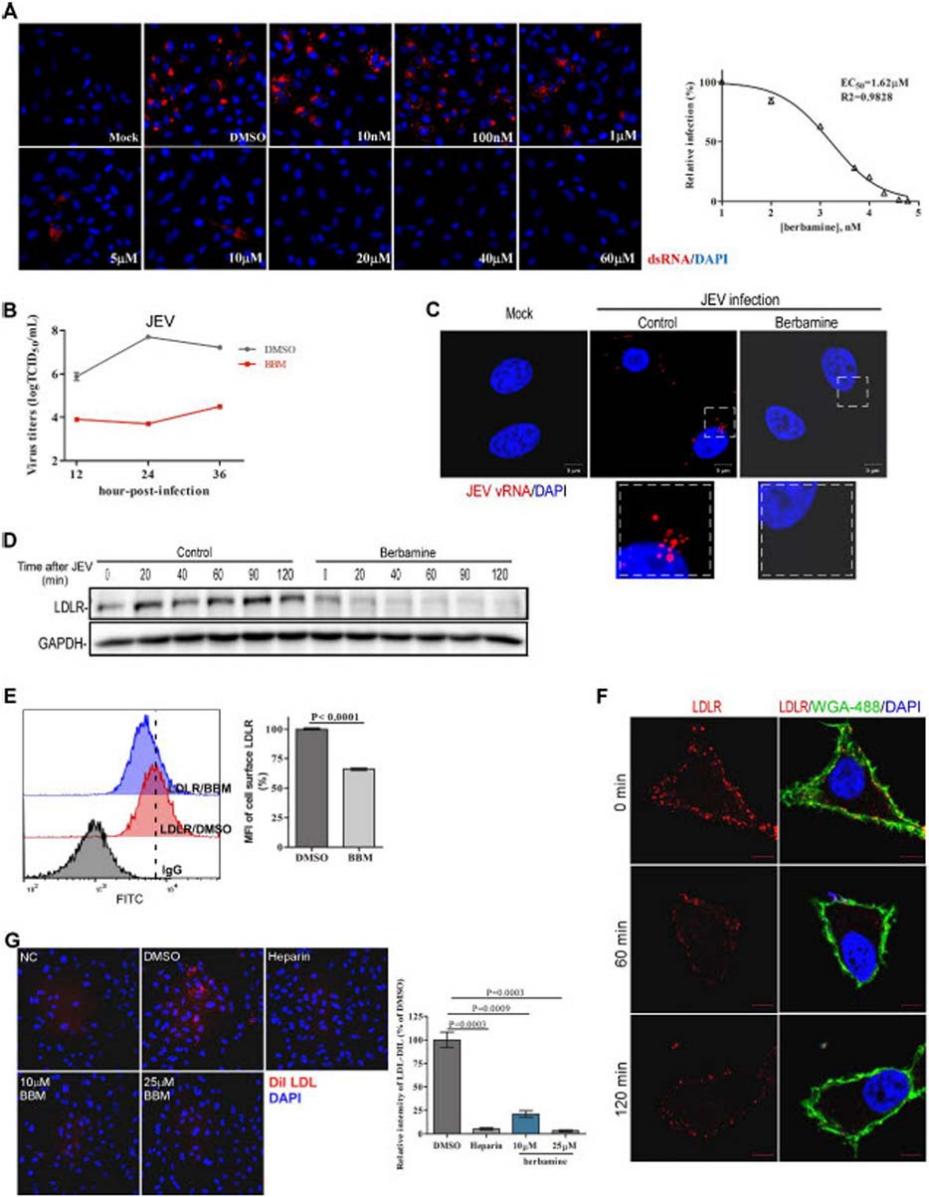
**Berbamine blocks JEV infection by depleting cell-surface LDLR.** (**A**) A549 cells were treated with DMSO or berbamine at different concentrations for 1 h, and then infected with ∼1 MOI JEV for 48 h. The cells were immunolabeled with dsRNA antibodies and subjected to fluorescence imaging. (**B**) Pretreatment of A549 cells with berbamine (BBM, 40 μM) significantly inhibited JEV progeny virion production, as determined by virus titer measurements. (**C**) A549 cells were treated with berbamine (20 μM) for 1 h, and were then incubated with ∼50 MOI JEV on ice for another 1 h. Afterward, they were incubated in the medium at 37 ˚C for another 80 min followed by in situ RNA hybridization to detect the RNA genome of JEV (JEV-vRNA) (red). (**D**) A549 cells were treated with or without berbamine (50 μM) for 1 h, and then they were incubated with JEV on ice for another 1 h. Thereafter, the cells were incubated at 37 ˚C for the indicated times followed by LDLR immunoblot analysis. (**E**) A549 cells were treated with or without berbamine (50 μM) for 3 h, followed by LDLR immunostaining. Subsequently, FACS analysis was performed to measure the cell surface LDLR. (**F**) A549 cells were treated with/without berbamine (50 μM) for the indicated times, followed by LDLR immunostaining. Alexa Fluor 488-conjugated wheat germ agglutinin (WGA) was used to label the plasma membrane. (**G**) A549 cells were pretreated with Heparin (250 μg/ml) or berbamine (10 μM or 25 μM) for 3 h, followed by incubation with Dil LDL on ice for another 1 h. Then, the cells were incubated at 37 ˚C for 30 min to allow the internalization of Dil LDL. The blots, images, and graphs represent data from three independent experiments. The difference between the two groups was analyzed using a two-tailed Student's t-test, *P*<0.05 was considered statistically significant.

***Berbamine inhibits JEV infection by decreasing the cell-surface LDLR level.*** Berbamine is a bis-benzylisoquinoline alkaloid isolated from berberis (one traditional Chinese medicine), and has reported effects on Ca^2+^ signaling [19–25]. We found that berbamine significantly inhibited JEV infection in A549 cells, and the half-maximal effective concentration (EC_50_) of berbamine against JEV is approximately 1.62 μM (Figure 2(A)). Consistently, berbamine significantly inhibited the infectious progeny viral particle production of JEV (Figure 2(B)). Moreover, berbamine blocked the entry of JEV into host cells (Figure 2(C)).

Interestingly, we found that JEV infection induced the LDLR accumulation, whereas berbamine treatment abolished this increase (Figure 2(D)). We, thus, examined whether berbamine decreases the LDLR levels at the cell surface by performing the LDLR immunostaining in cells treated with or without berbamine, followed by flow cytometric analysis or confocal imaging. We showed that berbamine significantly decreased the levels of LDLR at the plasma membrane (Figures 2(E and F)). In addition, we found that pretreatment of cells with berbamine markedly inhibited the uptake of Dil LDL, similar to the effect of heparin on uptake of Dil LDL (Figure 2(G)). In summary, these results suggest that berbamine might inhibit JEV infection by decreasing the level of LDLR at the plasma membrane.

LDLR is a known receptor for VSV [15]. We, thus, generated the lentivirus-based pseudotyped particles that incorporated VSV G protein and expressed a reporter gene (RFP-tagged Histone B), to assess whether berbamine also inhibits the entry of the VSV-G pseudotyped particles. As showed in **Figure S1**, pretreatment of cells with berbamine for 1.5 h effectively inhibited the entry of VSV-G pseudotyped particles into host cells, whereas treatment of cells with berbamine at 1.5 h post-infection had negligible effects on VSV entry.

***Berbamine inhibits endolysosomal trafficking and induces the secretion of extracellular vesicles to decrease cell-surface LDLR.*** We next investigated the mechanism by which berbamine decreases LDLR levels at the cell surface. We first examined whether berbamine regulates the general endolysosomal trafficking of cell surface receptors by performing the classical epidermal growth factor receptor (EGFR) degradation assay. As shown in **Figures S2A** and **S2B**, the fluorescently labeled epidermal growth factor (EGF-488)-EGFR complex entered cells and translocated to early endosomes and late endosomes/lysosomes in both DMSO- and berbamine-treated cells by 30 min to 1 h after EGF addition. At 3 h after EGF treatment, nearly all EGF-488-EGFR complex was degraded in control, not in berbamine-treated, cells. This data suggests that berbamine inhibits the endolysosomal degradation of EGFR but does not affect the internalization of the receptors.

**Figure 3.**
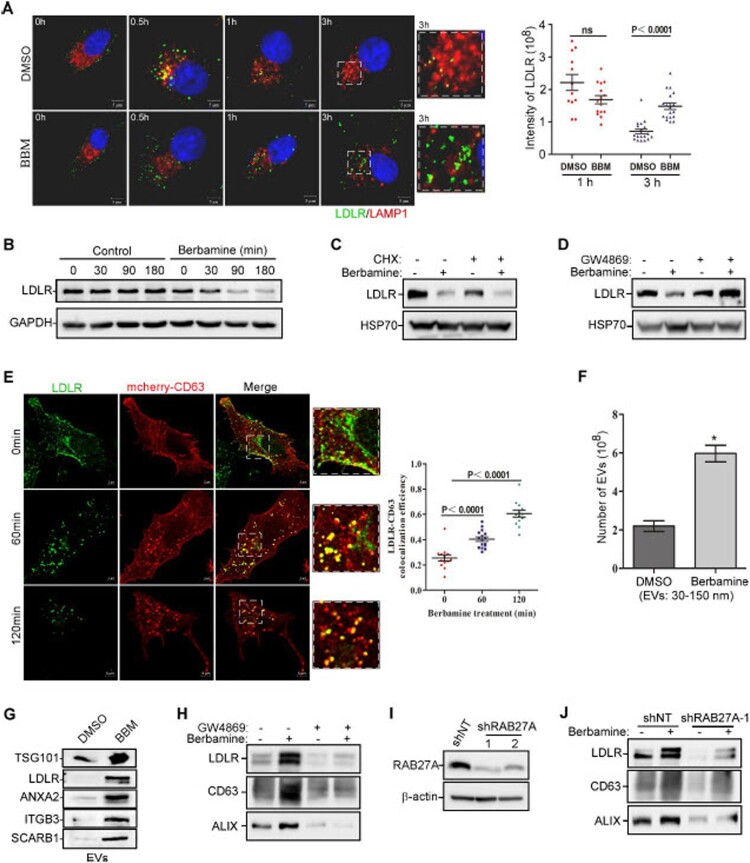
**Berbamine inhibits endolysosomal trafficking and induces EV secretion to decrease the cell-surface LDLR.** (**A**) A549 cells were treated with/without berbamine (50 μM) for 1 h, after which they were immunolabeled with an anti-LDLR primary antibody and the secondary antibody on ice. Afterward, cells were incubated at 37 ˚C for the indicated times, followed by LAMP1 immunostaining. (**B**) A549 cells were treated with/without berbamine (50 μM) for the indicated times, and cell lysates were then subjected to LDLR immunoblotting analysis. (**C, D**) A549 cells were pretreated with/without cycloheximide (CHX) (5 μg/ml) (**C**) or GW4869 (10 μM) (**D**) for 6 h, and were then treated with/without berbamine (50 μM) for another 3 h, followed by LDLR immunoblotting analysis. (**E**) CD63-mcherry-expressing A549 cells were treated with berbamine (50 μM) for the indicated times, and cells were then fixed and subjected to LDLR immunostaining. (**F**, **G**) EVs were collected from the culture medium of control or berbamine-treated (25 μM) A549 cells, and their concentration and distribution of sizes were determined with a nanoparticle tracking analyzer (**F**). The levels of TSG101, LDLR, ANXA2, ITGB3, and SCARB1 in these EVs were determined by immunoblot analysis (**G**). (**H**) EVs were collected from the culture medium of control or berbamine-treated A549 cells (25 μM) in the presence or absence of GW4689 (10 μM). The levels of LDLR, CD63, and ALIX in these EVs were determined by immunoblot analysis. (**I**) Immunoblot analysis of RAB27A was performed to confirm the knockdown efficiency of RAB27A shRNAs in A549 cells. (**J**) EVs were collected from the culture medium of control- and RAB27A-knockdown A549 cells treated with or without berbamine (25 μM). The levels of LDLR, CD63, and ALIX in these EVs were determined by immunoblot analysis. The blots, images, and graphs represent data from three independent experiments. The difference between the two groups was analyzed using a two-tailed Student's t-test, *P*<0.05 was considered statistically significant.

We then assessed whether berbamine affects the endolysosomal trafficking of LDLR by performing LDLR and LAMP1 co-immunostaining in cells treated with or without berbamine. In brief, A549 cells were first incubated with an anti-LDLR antibody on ice for 90 min. The internalization of the LDLR-antibody complex was initiated when cells were warmed to 37 ˚C [26]. In the control cells, after ∼30 min to 1 h, the internalized LDLR-antibody complex was found in the late endosomes or lysosomes, as shown by the colocalization between LDLR and LAMP1; and by ∼3 h, most internalized LDLR was degraded (top panel in Figure 3(A)**)**. However, in berbamine-treated cells, the LDLR-antibody complex was internalized normally but failed to be sent to lysosomes for degradation (bottom panel in Figure 3(A)).

Since the endolysosomal degradation of LDLR was compromised in the berbamine-treated cells (Figure 3(A)), we reasoned that this should lead to an increase in the total amount of LDLR in these cells. However, we found that berbamine markedly decreased the total amount of LDLR in cells (Figure 3(B)). In addition, berbamine decreased the level of LDLR in cells treated with or without cycloheximide **(**Figure 3(C)**)**, suggesting that the decrease of LDLR by berbamine is not due to the protein synthesis inhibition. Thus, we speculated that the reduced levels of LDLR that occurred in berbamine-treated cells might be due to an increase in the secretion of LDLR-containing EVs out of cells. To verify this possibility, we examined whether the blockage of EV secretion could reverse the decrease of LDLR induced by berbamine. As shown in Figure 3(D), GW4869, a sphingomyelinase inhibitor that abolishes the secretion of exosome [27,28], markedly reversed the decrease of LDLR induced by berbamine. This data suggests that berbamine might promote LDLR secretion through exosome, thereby decreasing the levels of LDLR. Therefore, we studied whether berbamine could induce the colocalization between LDLR and multivesicular body (MVB). As expected, upon berbamine treatment, LDLR gradually translocated from the plasma membrane to MVB, manifested by the increased colocalization between LDLR and CD63, a MVB marker (Figure 3(E)**).** Taken together, these results suggest that berbamine induces LDLR secretion via exosomes.

Dysregulated endolysosomal trafficking could promote the exosome release [29]. Therefore, we quantified the concentration of EVs in the cell culture medium of control or berbamine-treated cells using a nanoparticle analyzer. As expected, berbamine significantly promoted the secretion of EVs **(**Figure 3(F)**)**. We then examined whether these EVs contain elevated levels of membrane receptors in the berbamine-treated group when compared with the control group. Thus, EVs in the culture medium from the control or berbamine-treated cells were collected by ultracentrifugation, and the protein levels of LDLR and several other previously reported cell membrane receptors or membrane-binding proteins for viruses (e.g. ITGB3 [30–34], SCARB1 [35,36], and ANXA2 [37–39]), were analyzed by immunoblot analysis. We showed that the levels of LDLR, ANXA2, ITGB3, and SCARB1, similar to TSG10 (which is an exosome surface protein marker), were all markedly increased in EVs collected from the berbamine-treated cells when compared with the control group (Figure 3(G)). Moreover, GW4869 or RAB27A blocked the ability of berbamine to induce exosomal LDLR levels (Figure 3(H–J). Taken together, these results suggest that berbamine inhibits the endolysosomal trafficking of LDLR and/or other viral receptors. This leads to an increase in the level of exosomal LDLR and a concomitant decrease in its level at the plasma membrane.

**Figure 4.**
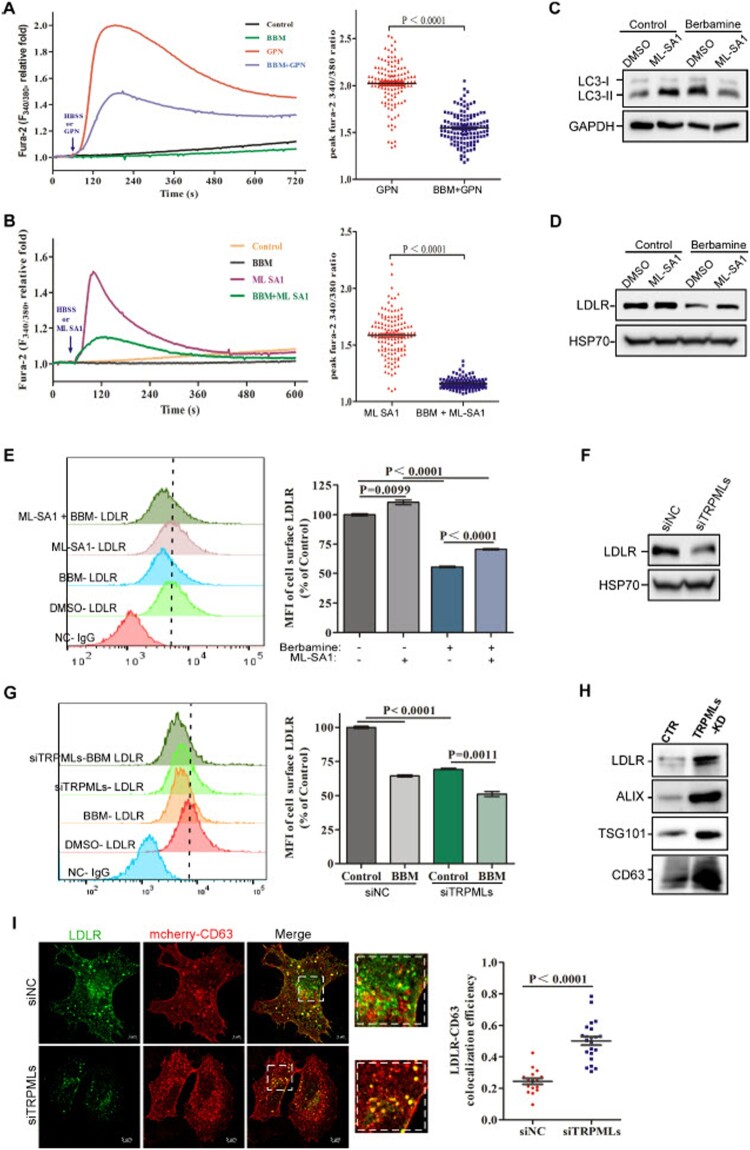
**Berbamine inhibits TRPMLs to promote LDLR-containing EV secretion.** (**A**, **B**) Berbamine significantly inhibited GPN- (**A**) or ML-SA1- (**B**) induced cytosolic Ca^2+^ increase in Fura-2-loaded HeLa cells. (**C**) A549 cells in the presence or absence of ML-SA1 (pretreated for 6h) were treated with or without berbamine for 3 h, followed by LC3B immunoblot analysis. (**D**, **E**) A549 cells in the presence or absence of ML-SA1 (pretreated for 6h) were treated with or without berbamine (50 μM) for 3 h, followed by LDLR immunoblot analysis (**D**); alternatively, the live cells were stained with the anti-LDLR antibody, followed by FACS analysis to measure the cell surface LDLR levels (**E**). (**F**) A549 cells were transfected with siRNAs against non-target control (siNC) or TRPMLs (siTRPML1-3), and cell lysates were subjected to LDLR immunoblot analysis. (**G**) A549 cells were transfected with siNC or siTRPMLs, and were then treated with berbamine (50 μM) for 3 h, followed by FACS analysis to measure the cell surface LDLR levels. (**H**) EVs were collected from the culture medium of control or TRPMLs-knockdown A549 cells, the levels of LDLR, CD63 and ALIX in these EVs were determined by immunoblot analysis. (**I**) CD63-mcherry was transiently transfected into control or TRPMLs-knockdown A549 cells. At 36h post-transfection, cells were fixed and subjected to LDLR immunostaining and confocal analysis. The blots, images, and graphs represent data from three independent experiments. The difference between the two groups was analyzed using a two-tailed Student's t-test, *P*<0.05 was considered statistically significant.

***Berbamine blocks lysosomal TRPMLs to promote the secretion of LDLR-containing exosomes.*** Although berbamine is known to be a calcium channel blocker, berbamine treatment did not change basal cytosolic Ca^2+^ concentration, nor affected thapsigargin (a specific Sarco/endoplasmic reticulum Ca^2+^-ATPase inhibitor)-induced Ca^2+^ release from ER, suggesting that berbamine does not affect ER Ca^2+^ pool. It also had little effect on store-operated Ca^2+^ entry (SOCE) (**Fig. S3**). Thus, we speculated that berbamine might inhibit endolysosomal trafficking by blocking the lysosomal calcium channels. We first examined whether berbamine affects the lysosomal Ca^2+^ levels by assessing the ability of Gly-Phe β-naphthylamide (GPN) to trigger Ca^2+^ release from lysosomes in cells treated with or without berbamine. We showed that berbamine significantly mitigated the GPN-induced cytosolic Ca^2+^ increase, suggesting that it inhibits the lysosomal Ca^2+^ channels (Figure 4(A)). Since TRPMLs in lysosomes and endosomes play critical roles in membrane trafficking, autophagy, and exocytosis [40–42], we further assessed whether berbamine blocks lysosomal Ca^2+^ release by inhibiting TRPMLs. As shown in Figure 4(B), berbamine significantly decreased the TRPML-mediated Ca^2+^ release from lysosomes, which was triggered by ML-SA1, a selective and potent TRPMLs agonist [43]. In addition, we found that pretreatment of ML-SA1 reversed the LC3-II accumulation induced by berbamine (Figure 4(C)), suggesting that berbamine blocks TRPMLs to impair lysosome function. Consistently, ML-SA1 reversed the LDLR decrease caused by berbamine (Figure 4(D and E)). These results again suggested that berbamine inhibits TRPMLs to block endolysosomal trafficking of LDLR. Subsequently, we knocked down the expression of TRPML1, 2, and 3 simultaneously by pools of siRNAs in A549 cells (Figure S4) and found that the intracellular and cell surface levels of LDLR were markedly decreased in TRPMLs-knockdown cells when compared to control cells (Figure 4(F and G)). Notably, the extent of decrease of LDLR levels at cell surface induced by berbamine in TRPMLs-knockdown cells was lower when compared to its effects on the control cells (Figure 4(G)). These results suggest that berbamine inhibits TRPMLs to reduce the levels of LDLR at the cell surface. In addition, we compared the amount of LDLR and exosome markers (TSG101, CD63 and ALIX) in the exosome isolated from control or TRPMLs-knockdown cells. We showed that TRPMLs knockdown markedly increased exosomal LDLR levels, along with exosome markers (Figure 4(H)). Moreover, we found that the colocalization between LDLR and CD63 was increased significantly in TRPMLs-knockdown cells when compared to control cells (Figure 4(I)). Taken together, these results suggest that berbamine inhibits TRPMLs to cause lysosomal dysfunction, and this, on one hand, inhibits the fusion between LDLR-containing MVBs/late endosomes and lysosomes to prevent the trafficking of LDLR, and on the other hand, promotes the fusion between MVBs and plasma membrane to increase exosome secretion.

**Figure 5.**
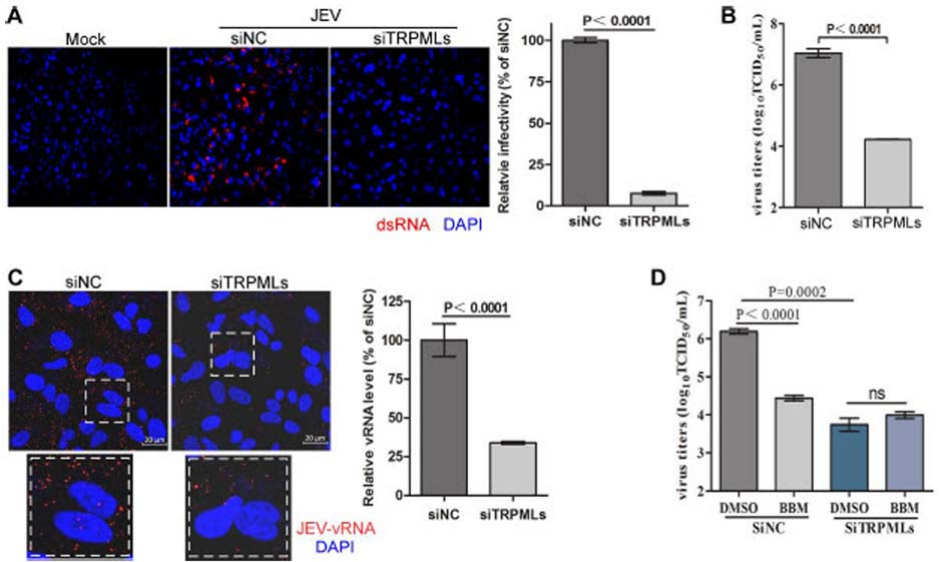
**Berbamine inhibits JEV infection through blockage of TRPMLs.** (**A, B**) A549 cells were transfected with siRNAs against non-target control (siNC) or TRPMLs (siTRPML1-3), and then cells were infected with ∼1MOI of JEV for 48 h or 24 h, followed by immunostaining against dsRNA (**A**) or titer measurement (**B**), respectively. (**C**) A549 cells were transfected with siNC or siTRPMLs, and were then infected with ∼50 MOI JEV for 90 min followed by *in situ* RNA hybridization to detect the RNA genome (red) of JEV. (**D**) A549 cells were transfected with siNC or siTRPMLs, then cells were pretreated with DMSO or berbamine (50 μM) for 3h, followed by infection with 10 MOI JEV. At 1.5 h post-infection, cell lysates were collected and subjected to titer measurement after 12 h. The images, and graphs represent data from three independent experiments. The difference between the two groups was analyzed using a two-tailed Student's t-test, *P*<0.05 was considered statistically significant.

***Berbamine inhibits JEV infection by blocking endolysosomal TRPMLs.*** We then investigated the role of TRPMLs in JEV infection. We showed that TRPMLs knockdown significantly inhibited JEV propagation (Figure 5(A and B)) and the entry of JEV into host cells (Figure 5(C)). Moreover, control- or TRPMLs-knockdown cells were pretreated with DMSO or berbamine for 3 h, and the culture medium containing berbamine and JEV were removed 1.5 h post-infection. Berbamine failed to further inhibit JEV infection in TRPMLs-knockdown cells (Figure 5(D)**)**. In summary, these results indicate that berbamine compromises the endolysosomal trafficking of LDLR via inhibition of TRPMLs, and this leads to a decrease in the levels of LDLR, thereby preventing JEV from entering the host cells.

It has been previously reported that tetrandrine (an analogue of berbamine) prevents the entry of the Ebola virus into host cells by blocking two-pore channels (TPCs) [44]. TPCs have also been shown to mediate MERS-CoV pseudovirus translation [45]. TPCs are Ca^2+^-permeable non-selective cation channels in the endo-lysosomal system [46,47]. We, thus, knocked down the expression of TPC1 or TPC2 in A549 cells (**Fig. S5A**) and found that knockdown of either TPC2 or TPC1 had little effect on JEV infection in A549 cells (**Figs S5B** and **S5C**). In addition, double knockdown of both TPC1 and TPC2 failed to inhibit JEV infection in A549 cells (**Fig. S5D**). These data indicate that berbamine does not target TPCs to inhibit JEV infection.

**Figure 6.**
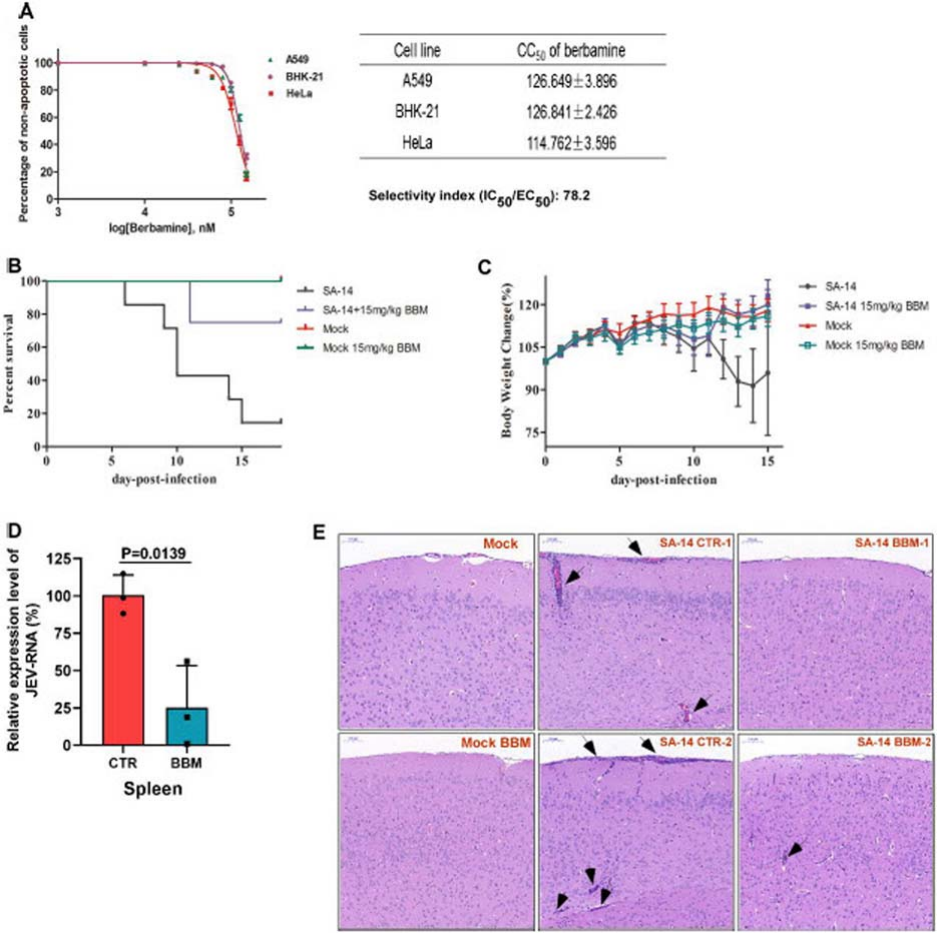
**Berbamine protects mice from lethal challenge of JEV.** (**A**) Cells grown in 96-well plates were treated with different concentrations of berbamine for 48 h, and then they were subjected to PI and Hoechst staining. PI-positive cells were quantified, and the CC_50_ values of berbamine in different cell lines were calculated. (**B-E**) Mice infected with the 10^7^ TCID_50_ SA-14 JEV strain were treated with or without 15 mg/kg berbamine (IP) twice per day for 15 days. Mice treated with berbamine without infection were used as a control to monitor the side effects of berbamine. The survival rate (**B**) and the bodyweight change of mice (**C**) were monitored every day. In addition, on Day 5 post-infection, the spleen and brain were collected, and were subjected to the viral load quantification (qRT-PCR) (**D**) and H&E staining (**E**), respectively. Arrows indicate histopathological changes, including meningitis, perivascular cuffing, and glial nodules. The images and graphs represent data from two independent experiments. The difference between the two groups was analyzed using a two-tailed Student's t-test, *P*<0.05 was considered statistically significant.

***Berbamine protects mice from the lethal challenge of JEV.*** We assessed the cytotoxicity of berbamine (Figure 6(A)) in different cell lines, and found that the half-maximal cytotoxicity concentration (CC_50_) for berbamine in these cell lines ranged from ∼115 μM to ∼127 μM (Figure 6(A)). We then calculated the selectivity index (SI) of berbamine for JEV infection. The SI helps to determine the window between cytotoxicity and antiviral activity by dividing the EC_50_ over its CC_50_ value (i.e. CC_50_/EC_50_). The SI value of berbamine is about 78, suggesting that berbamine is an anti-JEV agent with good therapeutic window. We, thus, assessed the protective effects of berbamine against JEV infection in a mouse model. As shown in Figure 6(**B** and **C)**, berbamine (15 mg/kg, IP, twice per day) protected mice from a lethal challenge of JEV, as demonstrated by the higher survival rate (i.e. 75% in the berbamine-treated group *versus* 12.5% in the control group) and the better bodyweight recovery. The virus shedding in the spleen of the berbamine-treated group (n=3) is much lower than in the control group (n=3) (Figure 6(D)). Moreover, berbamine alleviated the brain damage caused by JEV infection, such as meningitis, perivascular cuffing, vacuolar degeneration, and glial nodules, when comparing to the control group (Figure 6(E)). In summary, these results indicate that berbamine is a potential anti-JEV drug.

## Discussion

LDLR is the first member of the LDLR family to be identified, and the family also contains VLDLR, ApoER2, LRP1, LRP2, and LRP6. These family members all share several structural domains, such as LDLR repeats (for ligand binding), an EGF-like domain, and a transmembrane anchor motif. The LDLR family members mainly participate in lipoprotein trafficking to maintain cholesterol homeostasis [48]. LDLR has been shown to be one of the receptors for the hepatitis C virus, rhinovirus, and vesicular stomatitis virus [13–15,17]. LDLR is the receptor for low-density lipoprotein (LDL), and these receptor–ligand complexes are transported into the cells by clathrin-mediated endocytosis. Since LDLRs are internalized and recycle back to the plasma membrane every 10 min, they are ideal virus entry ports [11,12]. We found that LDLR knockdown significantly inhibited JEV infection (Figure 1(B-C)), specifically the entry into host cells. In addition, pre-incubation of cells with JEV impaired LDL uptake in a dose-dependent manner (Figure 1(E)). The direct interaction between LDLR and the JEV-E was confirmed by co-IP studies (Figure 1(I-IM)). The DIII of flavivirus Envelope protein has been shown to be the major antigenic domain and be responsible for receptor binding. However, we found that LDLR interacts with the DI-II of JEV-E, indicating that LDLR might be a cofactor facilitating internalization of the JEV-receptor complex, instead of a direct JEV receptor. Additional experiments are needed to confirm the exact role of LDLR in JEV entry. It is also of interest to study if other LDLR family members might be involved in JEV infection since they share a similar ligand-binding domain.

A number of studies have shown that virus infection changes the cytosolic Ca^2+^ homeostasis (or the resultant Ca^2+^ signaling) in the host cells, not only to facilitate the entry, replication, packaging, and release of the virus, but also to inhibit the cellular immune response against virus infection [44,49–53]. Here, we demonstrated that berbamine inhibited TRPML-mediated Ca^2+^ release from lysosomes to compromise the endolysosomal trafficking of host factors, e.g. membrane receptors for JEV, thereby promoting the secretion of these trapped host factors out of cells via EVs and decreasing the LDLR on the cell membrane (Figures 2–5). The decreased levels of viral host factors, such as LDLR, at the plasma membrane, are likely responsible for the anti-JEV activity of berbamine. Notably, berbamine did not change the functional SOCE (**Fig. S3**), and this might explain why it was only minimally cytotoxic (Figure 6(A)).

Lysosomes play an essential role in various intracellular vesicle trafficking pathways, including endocytosis, phagocytosis, autophagy, and exocytosis [54–56]. Loss of lysosomal Ca^2+^ homeostasis leads to dysfunctional lysosomes, resulting in the accumulation of damaged macromolecules and impaired organelles [57–61]. TRPMLs, which contain three members, including TRPML1, TRPML2 and TRPML3 [62,63], are one of the major types of Ca^2+^-permeable channels in lysosomes and endosomes. Mutated TRPML1 genes are the cause of human mucolipidosis type IV disease (ML4), in which abnormal lysosomal accumulation of lipids together with water-soluble substances are found in cells isolated from ML4 patients [64–66]. Here, we found that berbamine inhibited the ML-SA1 (a TRPML selective agonist)-induced release of Ca^2+^ from lysosomes (Figure 4(B)), and ML-SA1 reversed the reduced level of LDLR in berbamine-treated cells (Figure 4(D and E)). Moreover, both the intracellular level and cell surface level of LDLR were significantly decreased in TRPLMs-knockdown cells when compared to control cells (Figure 4(F-G)). The colocalization between LDLR and CD63 was increased significantly in TRPMLs-knockdown cells when compared to control cells (Figure 4(I)). These results suggest that berbamine blocks TRPMLs to inhibit the fusion between MVBs/late endosomes and lysosomes. The accumulation of MVBs likely leads to an increase in the fusion between MVBs and plasma membrane, and this results in the increased secretion of exosomes containing various host factors (e.g. LDLR), and a concomitant decrease in these factors at the plasma membrane, thereby rendering the cell resistant to JEV infection. As expected, TRPMLs knockdown significantly impaired JEV infection (Figure 5(A–C)). Moreover, berbamine did not further inhibit JEV infection in TRPMLs-KD cells at the early stage of the JEV life cycle (Figure 5(D)).

Interestingly, treating the A549 cells with berbamine after 1 h of JEV infection still significantly inhibited JEV propagation (**Fig. S6**). This suggests that berbamine not only inhibits the entry of JEV into host cells but also affects other stages of the viral life cycle. Notably, it has been shown that berbamine is an autophagy inhibitor via the blockage of SNARE-mediated autophagosome-lysosome fusion [67]. Actually, several late-stage autophagy inhibitors, such as chloroquine and bafilomycin A1, markedly inhibit flavivirus infections [68–72]. Thus, berbamine might impair the post-entry stages of JEV infection by blocking autophagosome-lysosome fusion. The underlying mechanism of how berbamine might affect JEV replication via the blockage of autophagy remains to be determined.

Berbamine is also known to be an ATP-competitive inhibitor of Ca^2+^/calmodulin-dependent protein kinase II (CaMKII), and it inhibits CaMKII to destabilize c-Myc, an oncoprotein, thereby reducing tumor burden [73,74]. CAMK2D is the dominant CaMKII family member in A549 cells, and we knocked down the expression of CAMK2D in A549 cells (**Fig. S7A)**. However, CAMK2D knockdown only subtly inhibited JEV infection (**Figs S7B** and **S7C**). This result indicates that CaMKII is not essential for JEV replication, and it is not the primary target for berbamine’s impairment of JEV infection at post-entry stages.

Berbamine is widely used to treat leukopenia in China and Japan for many years [75–77]. Berbamine also exhibits anti-inflammatory, immunosuppressive, anti-arrhythmia, anti-myocardial ischemia, anti-hypertension, and antithrombosis activities [21,78]. We showed that berbamine potently inhibited the JEV infection *in vitro* (Figure 2(A and B)), and protected mice from the lethal challenge of JEV (Figure 6(B-D)). Therefore, berbamine is a potential and attractive therapeutic agent for the prevention and/or treatment of JEV infection.

## Abbreviations

Berbamine (BBM); Japanese encephalitis virus (JEV); Low-density lipoprotein receptor (LDLR); Transient receptor potential mucolipin (TRPML).

## References

[1] Heinz FX, Stiasny K. Flaviviruses and flavivirus vaccines. Vaccine. 2012;30:4301–4306. 22682286 10.1016/j.vaccine.2011.09.114

[2] Mackenzie JS, Gubler DJ, Petersen LR. Emerging flaviviruses: the spread and resurgence of Japanese encephalitis, West Nile and dengue viruses. Nat Med. 2004;10:S98–S109. 15577938 10.1038/nm1144

[3] Monath TP. (1990). Flaviviruses. (ARMY MEDICAL RESEARCH INST OF INFECTIOUS DISEASES FORT DETRICK MD).

[4] Solomon T, Mallewa M. Dengue and other emerging flaviviruses. J. Infect. 2001;42:104–115. 11531316 10.1053/jinf.2001.0802

[5] Campbell GL, Hills SL, Fischer M, et al. Estimated global incidence of Japanese encephalitis: a systematic review. Bull World Health Organ. 2011;89:766–774. 22084515 10.2471/BLT.10.085233 PMC3209971

[6] Gould GW, Lippincott-Schwartz J. New roles for endosomes: from vesicular carriers to multi-purpose platforms. Nat Rev Mol Cell Biol. 2009;10:287–292. 19277045 10.1038/nrm2652 PMC3690957

[7] Kowal J, Tkach M, Théry C. Biogenesis and secretion of exosomes. Curr Opin Cell Biol. 2014;29:116–125. 24959705 10.1016/j.ceb.2014.05.004

[8] McAndrews KM, Kalluri R. Mechanisms associated with biogenesis of exosomes in cancer. Mol Cancer. 2019;18:52. 30925917 10.1186/s12943-019-0963-9 PMC6441149

[9] Go G-w, Mani A. Low-density lipoprotein receptor (LDLR) family orchestrates cholesterol homeostasis. Yale J Biol Med. 2012;85:19–28. 22461740 PMC3313535

[10] Goldstein JL, Brown MS. Binding and degradation of Low density lipoproteins by cultured human fibroblasts comparison of cells from a normal subject and from a patient with homozygous familial hypercholesterolemia. J Biol Chem. 1974;249:5153–5162. 4368448

[11] Brown MS, Herz J, Goldstein JL. Calcium cages, acid baths and recycling receptors. Nature. 1997;388:629–630. 9262394 10.1038/41672

[12] Finkelshtein D, Werman A, Novick D, et al. LDL receptor and its family members serve as the cellular receptors for vesicular stomatitis virus. Proc Natl Acad Sci USA. 2013;110:7306–7311. 23589850 10.1073/pnas.1214441110 PMC3645523

[13] Monazahian M, Bohme I, Bonk S, et al. Low density lipoprotein receptor as a candidate receptor for hepatitis C virus. J Med Virol. 1999;57:223–229. 10022791 10.1002/(sici)1096-9071(199903)57:3<223::aid-jmv2>3.0.co;2-4

[14] Wunschmann S, Medh JD, Klinzmann D, et al. Characterization of hepatitis C virus (HCV) and HCV E2 interactions with CD81 and the low-density lipoprotein receptor. J Virol. 2000;74:10055–10062. 11024134 10.1128/jvi.74.21.10055-10062.2000 PMC102044

[15] Finkelshtein D, Werman A, Novick D, et al. LDL receptor and its family members serve as the cellular receptors for vesicular stomatitis virus. Proc Natl Acad Sci U S A. 2013;110:7306–7311. 23589850 10.1073/pnas.1214441110 PMC3645523

[16] Fischer DG, Tal N, Novick D, et al. An antiviral soluble form of the LDL receptor induced by interferon. Science. 1993;262:250–253. 8211145 10.1126/science.8211145

[17] Bochkov YA, Gern JE. Rhinoviruses and their receptors: implications for allergic disease. Curr Allergy Asthma Rep. 2016;16:30. 26960297 10.1007/s11882-016-0608-7 PMC4854667

[18] Wei J-c, Huang Y-z, Zhong D-k, et al. Design and evaluation of a multi-epitope peptide against Japanese encephalitis virus infection in BALB/c mice. Biochem Biophys Res Commun. 2010;396:787–792. 20457131 10.1016/j.bbrc.2010.04.133

[19] Zhang CM, Gao L, Zheng YJ, et al. Berbamine protects the heart from ischemia/reperfusion injury by maintaining cytosolic Ca(2+) homeostasis and preventing calpain activation. Circ J. 2012;76:1993–2002. 22664727 10.1253/circj.cj-11-1431

[20] Hu H, Zhou S, Sun X, et al. A potent antiarrhythmic drug N-methyl berbamine extends the action potential through inhibiting both calcium and potassium currents. J Pharmacol Sci. 2020;142:131–139. 31992491 10.1016/j.jphs.2019.12.008

[21] Guo ZB, Fu JG. [Progress of cardiovascular pharmacologic study on berbamine]. Zhongguo Zhong Xi Yi Jie He Za Zhi. 2005;25:765–768. 16152843

[22] Li BY, Qiao GF, Zhao YL, et al. Effects of berbamine on ATP-induced [Ca2+]i mobilization in cultured vascular smooth muscle cells and cardiomyocytes. Zhongguo Yao Li Xue Bao. 1999;20:705–708. 10678102

[23] Qiao GF, Zhou H, Li BY, et al. Antagonistic effects of berbamine on [Ca2+]i mobilization by KCl, norepinephrine, and caffeine in newborn rat cardiomyocytes. Zhongguo Yao Li Xue Bao. 1999;20:292–296. 10452111

[24] Leung YM, Berdik M, Kwan CY, et al. Effects of tetrandrine and closely related bis-benzylisoquinoline derivatives on cytosolic Ca2+ in human leukaemic HL-60 cells: a structure-activity relationship study. Clin Exp Pharmacol Physiol. 1996;23:653–659. 8886484 10.1111/j.1440-1681.1996.tb01752.x

[25] Li BY, Zhang YC, Li WH. Effects of berbamine on contraction and Ca2+ influx of pig basilar artery. Zhongguo Yao Li Xue Bao. 1992;13:412–416. 1300042

[26] Powelka AM, Sun J, Li J, et al. Stimulation-dependent recycling of integrin beta1 regulated by ARF6 and Rab11. Traffic. 2004;5:20–36. 14675422 10.1111/j.1600-0854.2004.00150.x

[27] Chairoungdua A, Smith DL, Pochard P, et al. Exosome release of beta-catenin: a novel mechanism that antagonizes Wnt signaling. J Cell Biol. 2010;190:1079–1091. 20837771 10.1083/jcb.201002049 PMC3101591

[28] Kosaka N, Iguchi H, Yoshioka Y, et al. Secretory mechanisms and intercellular transfer of microRNAs in living cells. J Biol Chem. 2010;285:17442–17452. 20353945 10.1074/jbc.M110.107821 PMC2878508

[29] Ortega FG, Roefs MT, de Miguel Perez D, et al. Interfering with endolysosomal trafficking enhances release of bioactive exosomes. Nanomedicine. 2019;20:102014. 31152797 10.1016/j.nano.2019.102014

[30] Jackson T, Sharma A, Ghazaleh RA, et al. Arginine-glycine-aspartic acid-specific binding by foot-and-mouth disease viruses to the purified integrin alpha (v) beta3 in vitro. J Virol. 1997;71:8357–8361. 9343190 10.1128/jvi.71.11.8357-8361.1997 PMC192296

[31] Gianni T, Gatta V, Campadelli-Fiume G. αVβ3-integrin routes herpes simplex virus to an entry pathway dependent on cholesterol-rich lipid rafts and dynamin2. Proc Natl Acad Sci USA. 2010;107:22260–22265. 21135248 10.1073/pnas.1014923108 PMC3009828

[32] Chu JJ-h, Ng M-L. Interaction of West Nile virus with αvβ3 integrin mediates virus entry into cells. J Biol Chem. 2004;279:54533–54541. 15475343 10.1074/jbc.M410208200

[33] Chu J, Ng M. Characterization of a 105-kDa plasma membrane associated glycoprotein that is involved in West Nile virus binding and infection. Virology. 2003;312:458–469. 12919750 10.1016/s0042-6822(03)00261-7

[34] Berinstein A, Roivainen M, Hovi T, et al. Antibodies to the vitronectin receptor (integrin alpha V beta 3) inhibit binding and infection of foot-and-mouth disease 7virus to cultured cells. J Virol. 1995;69:2664–2666. 7533862 10.1128/jvi.69.4.2664-2666.1995 PMC188951

[35] Westhaus S, Deest M, Nguyen AT, et al. Scavenger receptor class B member 1 (SCARB1) variants modulate hepatitis C virus replication cycle and viral load. J Hepatol. 2017;67:237–245. 28363797 10.1016/j.jhep.2017.03.020

[36] Li Y, Kakinami C, Li Q, et al. Human apolipoprotein AI is associated with dengue virus and enhances virus infection through SR-BI. PloS one. 2013;8. 10.1371/journal.pone.0070390 PMC3722190 23894648

[37] Raynor CM, Wright JF, Waisman DM, et al. Annexin II enhances cytomegalovirus binding and fusion to phospholipid membranes. Biochemistry. 1999;38:5089–5095. 10213612 10.1021/bi982095b

[38] Mei M, Ye J, Qin A, et al. Identification of novel viral receptors with cell line expressing viral receptor-binding protein. Sci Rep. 2015;5:7935. 25604889 10.1038/srep07935 PMC4300512

[39] Gonzalez-Reyes S, García-Manso A, del Barrio G, et al. Role of annexin A2 in cellular entry of rabbit vesivirus. J Gen Virol. 2009;90:2724–2730. 19605586 10.1099/vir.0.013276-0

[40] Wang W, Zhang X, Gao Q, et al. TRPML1: an ion channel in the lysosome. Handb Exp Pharmacol. 2014;222:631–645. 24756723 10.1007/978-3-642-54215-2_24

[41] Venkatachalam K, Wong CO, Zhu MX. The role of TRPMLs in endolysosomal trafficking and function. Cell Calcium. 2015;58:48–56. 25465891 10.1016/j.ceca.2014.10.008 PMC4412768

[42] Cheng X, Shen D, Samie M, et al. Mucolipins: intracellular TRPML1-3 channels. FEBS Lett. 2010;584:2013–2021. 20074572 10.1016/j.febslet.2009.12.056 PMC2866799

[43] Feng X, Xiong J, Lu Y, et al. Differential mechanisms of action of the mucolipin synthetic agonist, ML-SA1, on insect TRPML and mammalian TRPML1. Cell Calcium. 2014;56:446–456. 25266962 10.1016/j.ceca.2014.09.004 PMC4252876

[44] Sakurai Y, Kolokoltsov AA, Chen C-C, et al. Two-pore channels control Ebola virus host cell entry and are drug targets for disease treatment. Science. 2015;347:995–998. 25722412 10.1126/science.1258758 PMC4550587

[45] Gunaratne GS, Yang Y, Li F, et al. NAADP-dependent Ca(2+) signaling regulates Middle East respiratory syndrome-coronavirus pseudovirus translocation through the endolysosomal system. Cell Calcium. 2018;75:30–41. 30121440 10.1016/j.ceca.2018.08.003 PMC6251489

[46] Grimm C, Chen CC, Wahl-Schott C, et al. Two-Pore channels: catalyzers of endolysosomal transport and function. Front Pharmacol. 2017;8:45. 28223936 10.3389/fphar.2017.00045 PMC5293812

[47] Patel S. Function and dysfunction of two-pore channels. Sci Signal. 2015;8:re7. 26152696 10.1126/scisignal.aab3314

[48] Go GW, Mani A. Low-density lipoprotein receptor (LDLR) family orchestrates cholesterol homeostasis. Yale J Biol Med. 2012;85:19–28. 22461740 PMC3313535

[49] Zhou Y, Frey TK, Yang JJ. Viral calciomics: interplays between Ca2+ and virus. Cell Calcium. 2009;46:1–17. 19535138 10.1016/j.ceca.2009.05.005 PMC3449087

[50] Scherbik SV, Brinton MA. Virus-induced Ca2+ influx extends survival of west Nile virus-infected cells. J Virol. 2010;84:8721–8731. 20538858 10.1128/JVI.00144-10 PMC2918993

[51] Haughey NJ, Mattson MP. Calcium dysregulation and neuronal apoptosis by the HIV-1 proteins Tat and gp120. J Acquir Immune Defic Syndr. 2002;31:S55–S61. 12394783 10.1097/00126334-200210012-00005

[52] Fujioka Y, Nishide S, Ose T, et al. A sialylated voltage-dependent Ca2+ channel binds hemagglutinin and mediates influenza A virus entry into mammalian cells. Cell Host Microbe. 2018;23:809–818. e805. 29779930 10.1016/j.chom.2018.04.015

[53] Bissig C, Lenoir M, Velluz M-C, et al. Viral infection controlled by a calcium-dependent lipid-binding module in ALIX. Dev Cell. 2013;25:364–373. 23664863 10.1016/j.devcel.2013.04.003 PMC4129370

[54] Saftig P, Klumperman J. Lysosome biogenesis and lysosomal membrane proteins: trafficking meets function. Nat Rev Mol Cell Biol. 2009;10:623–635. 19672277 10.1038/nrm2745

[55] Luzio JP, Rous BA, Bright NA, et al. Lysosome-endosome fusion and lysosome biogenesis. J Cell Sci. 2000;113:1515–1524. 10751143 10.1242/jcs.113.9.1515

[56] Blott EJ, Griffiths GM. Secretory lysosomes. Nat Rev Mol Cell Biol. 2002;3:122–131. 11836514 10.1038/nrm732

[57] Shen D, Wang X, Li X, et al. Lipid storage disorders block lysosomal trafficking by inhibiting a TRP channel and lysosomal calcium release. Nat Commun. 2012;3:1–11. 10.1038/ncomms1735 PMC3347486 22415822

[58] Pryor PR, Mullock BM, Bright NA, et al. The role of intraorganellar Ca2+ in late endosome–lysosome heterotypic fusion and in the reformation of lysosomes from hybrid organelles. J Cell Biol. 2000;149:1053–1062. 10831609 10.1083/jcb.149.5.1053 PMC2174832

[59] Morgan AJ, Platt FM, Lloyd-Evans E, et al. Molecular mechanisms of endolysosomal Ca2+ signalling in health and disease. Biochem J. 2011;439:349–378. 21992097 10.1042/BJ20110949

[60] Luzio J, Bright N, Pryor P. The role of calcium and other ions in sorting and delivery in the late endocytic pathway. Portland Press Ltd. 2007;35:1088–1091. 10.1042/BST0351088 17956286

[61] Christensen KA, Myers JT, Swanson JA. pH-dependent regulation of lysosomal calcium in macrophages. J Cell Sci. 2002;115:599–607. 11861766 10.1242/jcs.115.3.599

[62] Zeevi DA, Frumkin A, Bach G. TRPML and lysosomal function. Biochimica et Biophysica Acta (BBA)-Molecular Basis of Disease. 2007;1772:851–858. 17306511 10.1016/j.bbadis.2007.01.004

[63] Bach G. Mucolipin 1: endocytosis and cation channel—a review. Pflügers Archiv. 2005;451:313–317. 15570434 10.1007/s00424-004-1361-7

[64] Sun M, Goldin E, Stahl S, et al. Mucolipidosis type IV is caused by mutations in a gene encoding a novel transient receptor potential channel. Hum Mol Genet. 2000;9:2471–2478. 11030752 10.1093/hmg/9.17.2471

[65] Berman E, Livni N, Shapira E, et al. Congenital corneal clouding with abnormal systemic storage bodies: a new variant of mucolipidosis. J Pediatr. 1974;84:519–526. 4365943 10.1016/s0022-3476(74)80671-2

[66] Bargal R, Avidan N, Ben-Asher E, et al. Identification of the gene causing mucolipidosis type IV. Nat Genet. 2000;26:118–122. 10973263 10.1038/79095

[67] Zheng Y, Gu S, Li X, et al. Berbamine postconditioning protects the heart from ischemia/reperfusion injury through modulation of autophagy. Cell Death Dis. 2017;8:e2577–e2577. 28151484 10.1038/cddis.2017.7 PMC5386498

[68] Li C, Zhu X, Ji X, et al. Chloroquine, a FDA-approved drug, prevents Zika virus infection and its associated congenital microcephaly in mice. EBioMedicine. 2017;24:189–194. 29033372 10.1016/j.ebiom.2017.09.034 PMC5652284

[69] Cao B, Parnell LA, Diamond MS, et al. Inhibition of autophagy limits vertical transmission of Zika virus in pregnant mice. J Exp Med. 2017;214:2303–2313. 28694387 10.1084/jem.20170957 PMC5551583

[70] Baloch AS, Liu C, Liang X, et al. Avian flavivirus enters BHK-21 cells by a Low pH-dependent endosomal pathway. Viruses. 2019;11:1112. 10.3390/v11121112 PMC6949961 31801284

[71] Huang L, Fu Q, Dai J-M, et al. High-content screening of diterpenoids from isodon species as autophagy modulators and the functional study of their antiviral activities. Cell Biol Toxicol. 2021: 1–19. 10.1007/s10565-021-09580-6 33486680

[72] Xu Q, Huang L, Xing J, et al. Japanese encephalitis virus manipulates lysosomes membrane for RNA replication and utilizes autophagy components for intracellular growth. Vet Microbiol. 2021;255:109025. 33725516 10.1016/j.vetmic.2021.109025

[73] Gu Y, Zhang J, Ma X, et al. Stabilization of the c-Myc protein by CAMKIIγ promotes T cell lymphoma. Cancer Cell. 2017;32:115–128. e117. 28697340 10.1016/j.ccell.2017.06.001 PMC5552197

[74] Gu Y, Chen T, Meng Z, et al. CaMKII γ, a critical regulator of CML stem/progenitor cells, is a target of the natural product berbamine. blood. The Journal of the American Society of Hematology. 2012;120:4829–4839. 10.1182/blood-2012-06-434894 PMC4507036 23074277

[75] Chang-Xiao Liu PGX, Guo-Sheng L. Studies on plant resources, pharmacology and clinical treatment with berbamine. Phytother Res. 1991;5:228–230.

[76] MORI M, KAWASAKI S, SACHO M, et al. Effect of cepharanthin on the hemopoietic suppression by X-ray irradiation -- hematological studies (in Japanese). Gann to Kagakuryouhou. 1989;49:667–674. 2798058

[77] Oyaizu H, Adachi Y, Yasumizu R, et al. Protection of T cells from radiation-induced apoptosis by cepharanthin. Int Immunopharmacol. 2001;1:2091–2099. 11710538 10.1016/s1567-5769(01)00127-8

[78] Zhang W, Chen SG, Ju HS, et al. Mechanisms of protective effects of berbamine on ischemia/reperfusion injury in isolated rat heart. Methods Find Exp Clin Pharmacol. 1992;14:677–684. 1338220

